# The interplay between non-coding RNAs and alternative splicing: from regulatory mechanism to therapeutic implications in cancer

**DOI:** 10.7150/thno.83920

**Published:** 2023-04-23

**Authors:** Min Liu, Subo Zhang, Heng Zhou, Xiaoyun Hu, Jianing Li, Boshi Fu, Minjie Wei, Huilin Huang, Huizhe Wu

**Affiliations:** 1 Department of Pharmacology, School of Pharmacy, China Medical University, Shenyang, Liaoning 110122, P. R. China.; 2 Liaoning Key Laboratory of molecular targeted anti-tumor drug development and evaluation, Liaoning Cancer immune peptide drug Engineering Technology Research Center, Key Laboratory of Precision Diagnosis and Treatment of Gastrointestinal Tumors, Ministry of Education, China Medical University, Shenyang, Liaoning 110122, P. R. China.; 3 Sun Yat-sen University Cancer Center, State Key Laboratory of Oncology in South China, Collaborative Innovation Center for Cancer Medicine, Guangzhou, Guangdong, 510060, P. R. China.; 4 Department of Surgical Oncology and General Surgery, The First Hospital of China Medical University, Shenyang, Liaoning 110001, P. R. China.; 5 Shenyang Kangwei Medical Laboratory Analysis Co. LTD, Shenyang, Liaoning, P. R. China.

**Keywords:** Alternative splicing, Non-coding RNA, Cancer, Drug resistance, Chemotherapy, Targeted therapy, Immunotherapy

## Abstract

Alternative splicing (AS) is a common and conserved process in eukaryotic gene regulation. It occurs in approximately 95% of multi-exon genes, greatly enriching the complexity and diversity of mRNAs and proteins. Recent studies have found that in addition to coding RNAs, non-coding RNAs (ncRNAs) are also inextricably linked with AS. Multiple different types of ncRNAs are generated by AS of precursor long non-coding (pre-lncRNAs) or precursor messenger RNAs (pre-mRNAs). Furthermore, ncRNAs, as a novel class of regulators, can participate in AS regulation by interacting with the cis-acting elements or trans-acting factors. Several studies have implicated abnormal expression of ncRNAs and ncRNA-related AS events in the initiation, progression, and therapy resistance in various types of cancers. Therefore, owing to their roles in mediating drug resistance, ncRNAs, AS-related factors and AS-related novel antigens may serve as promising therapeutic targets in cancer treatment. In this review, we summarize the interaction between ncRNAs and AS processes, emphasizing their great influences on cancer, especially on chemoresistance, and highlighting their potential values in clinical treatment.

## Background

Gene expression is tightly regulated at multiple levels during physiological processes, whose dysregulation is associated with several diseases, including cancer 1. RNA splicing, a fundamental step of gene expression, involves the removal of introns from precursor messenger RNAs (pre-mRNAs) and the merging of the exons to form mature mRNAs 2-4. Alternative selection of spliced exons, also known as alternative splicing (AS), can result in different protein products from a single primary transcript, and thus AS acts as a predominant post-transcriptional regulatory mechanism of gene expression 5, 6.

In addition to coding RNAs, non-coding RNAs (ncRNAs) are also inextricably linked with AS. Non-coding RNAs (ncRNAs) are functional RNAs that do not encode proteins. The advancement in high-throughput sequencing in the last decades has allowed us to identify a large number of ncRNAs, including long non-coding RNAs (lncRNAs), microRNAs (miRNAs), circular RNAs (circRNAs), small nucleolar RNAs (snoRNAs) and small nuclear RNA (snRNAs), which are generated by AS of pre-lncRNAs or pre-mRNAs 7-11. Recent studies have shown that ncRNAs, originally considered as transcriptional “junks”, play vital roles in gene expression regulation, including AS 12-17. Extensive interactions between ncRNAs and AS have been reported, which contribute to the complexity of gene regulation in cancer. Therefore, deciphering their interplay would help to determine cancer pathogenesis of cancer and provide new insights into cancer therapy. In this review, we summarize the molecular mechanisms and potential roles of the interaction between ncRNAs and AS in the development, progression and multi-drug resistance (MDR) of various cancers and discuss the latest development in therapeutic strategies targeting AS or AS-related ncRNAs.

## The regulation mechanism of RNA splicing

**Figure 1:**
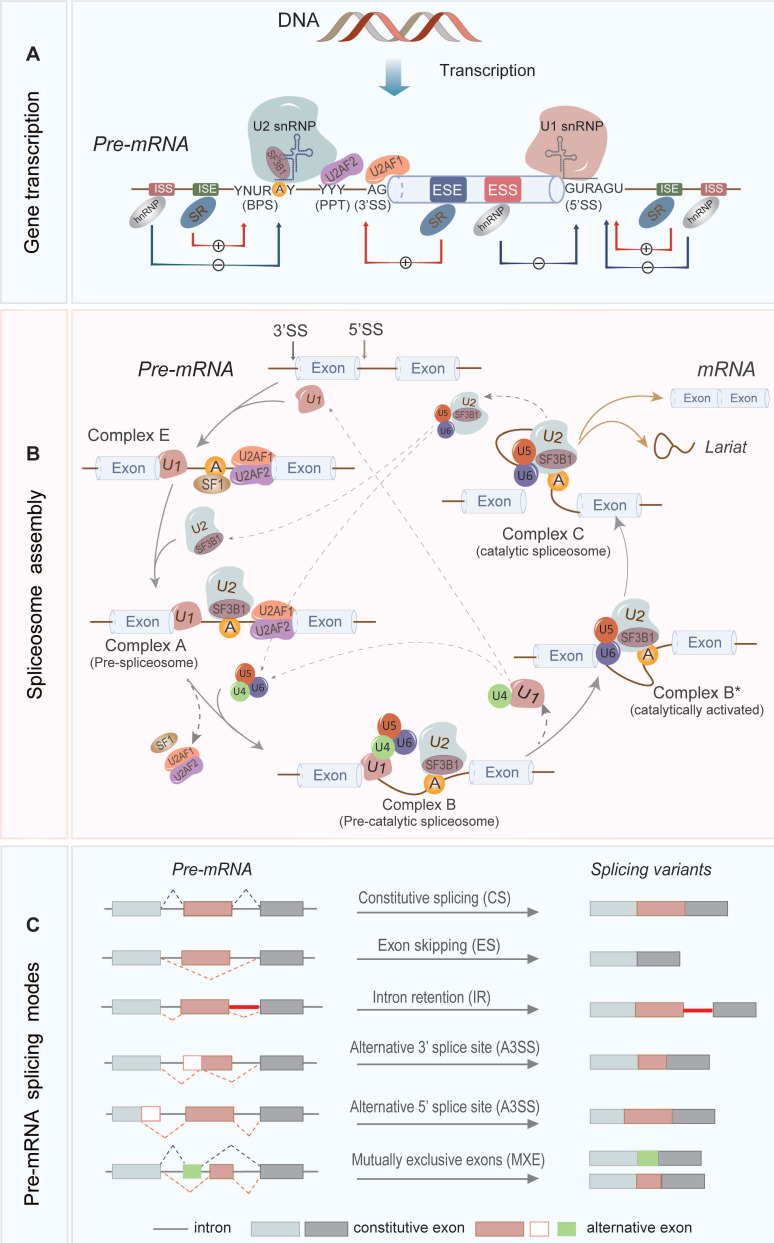
The regulation mechanism and mode of RNA splicing. **(A)** The cis-acting elements on the primary transcription product include a 5' splice site (5'SS), 3' splice site (3'SS), branch point site (BPS), polypyrimidine tract (PPT), and splicing regulatory elements (SREs) in the proximity of splice site. The SREs are subdivided into intronic and exonic splice enhancers and silencers (ISEs, ISSs, ESEs, and ESSs, respectively). SR proteins as splicing activators enhance the utilization of splice sites by preferentially combining with ESEs and ISEs; and conversely, hnRNPs as splicing repressors inhibit the binding to the splice sites by interacting with ESSs and ISSs. **(B)** U1 snRNP recognizes 5'SS and binds it via base pairing, while SF1, U2AF2 and U2AF1 combines separately the BPS, PPT and 3'SS, forming early complex E. Then U2 snRNP replaces SF1 and binds to the BPS to form complex A. The U4/U6-U5 tri- snRNP complex is subsequently recruited to form a pre-catalytic spliceosome. The complex B is rearranged to form catalytically activated complex B*, which catalyzes the first transesterification reaction to produce Complex C, followed by the second transesterification reaction. Lastly, exons are interlinked to form mature mRNA, and the introns are degraded rapidly as the lariat and snRNPs are recovered. **(C)** RNA splicing consists of constitutive splicing (CS) and alternative splicing (AS), and various AS modes are generated based on the multiple splice sites and ways of exon linking, including exon skipping (ES), alternative 5' or 3' splice site selection (A5SS or A3SS), mutually exclusive exons (MXE) and intron retention (IR).

Pre-mRNA splicing in the eukaryotic cell was first discovered in 1977 using an electron microscope 18-20. To date, our understanding of RNA splicing has been greatly improved. As seen in Figure 1, RNA splicing is a highly regulated process, which requires coordination between spliceosomes, cis-acting elements, and trans-acting proteins to remove introns from pre-mRNAs and merge the protein-coding exons to generate mature mRNAs 21.

The introns contain three important sites, the 5' splice site (5'SS), branch point site (BPS), and 3' splice site (3'SS), which are short conserved sequences (Figure 1A). In general, the RNA splicing process is a two-step transesterification reaction that begins with a nucleophilic attack at the 5' SS (also known as the splice donor) by a 2'-hydroxyl group of the BP adenosine. This reaction creates a cleaved 5' exon and a lariat structure containing the intron and the 3' exon. Subsequently, the 3'-hydroxyl group on the detached 5' exon attacks the 3' SS (also referred as the splice acceptor), which removes the intron and ligates the exons to produce a mature mRNA 22, 23.

In the splicing process, the generation of spliceosome plays an important function (Figure 1B). The spliceosome is a large multimeric ribonucleoprotein (RNP) complex which consists of five small nuclear ribonucleoproteins (snRNPs), including U1, U2, U4/U6, and U5. The U1 and U2 snRNPs recognize the 5'SS and the BP sequence, respectively, and functionally generate the pre-spliceosome. The pre-spliceosome then associates with the preassembled U4/U6/U5 tri-snRNP to form the fully assembled spliceosome to execute the splicing function 24-27.

In addition, some other important cis-acting elements on pre-mRNA such as intronic splice enhancers and silencers, exonic splice enhancers and silencers (ISEs, ISSs, ESEs, and ESSs, respectively) are important for the splicing regulation by recruitment of splicing factors such as serine/arginine-rich (SR) proteins and heterogenous nuclear ribonucleoproteins (hnRNPs) (Figure 1A). The SR proteins contain one or more RNA recognition motifs that bind to ESEs and ISEs on the pre-mRNA to recruit other SFs. The hnRNPs are generally more diverse in their RNA-binding domain and preferentially interact with the splicing silencers 28-30.

More than 95% of human genes undergo AS, which contributes to the diversity of RNA transcripts and protein products 31-33. Figure 1C illustrates different types of AS, including exon skipping (ES), intron retention (IR), alternative 5' or 3' splice site selection (A5SS or A3SS) and mutually exclusive exons (MXE) 15, 34. AS is regulated by cis-acting elements in pre-mRNAs and trans-acting factors, whose mutation or deregulation could lead to aberrant AS events and is usually associated with tumorigenesis.

## NcRNAs produced by RNA splicing

**Figure 2:**
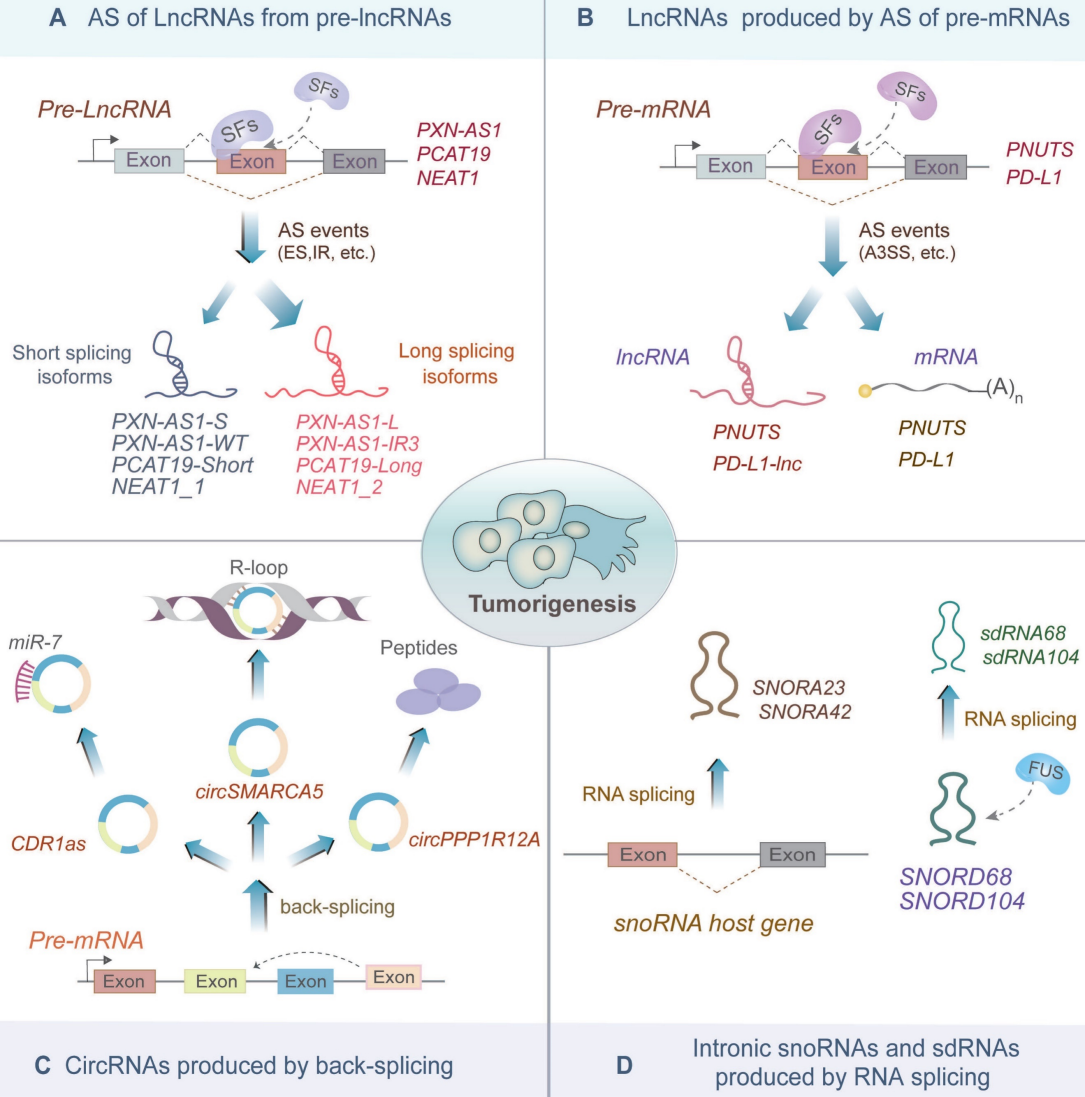
Non-coding RNAs produced by RNA splicing. **(A)** Precursor long non-coding RNAs (pre-lncRNAs) undergo splicing factor-mediated alternative splicing, which triggers the L/S switch. The long splicing isoforms, such as *PXN-AS1-L, PXN-AS1-IR3, PCAT19-Long* and *NEAT1_2*, have been reported to promote tumorigenesis. **(B)** LncRNAs *PNUTS* and *PD-L1-lnc* generated by bifunctional pre-mRNA splicing can promote carcinogenesis. **(C)** CircRNAs produced by backing splicing of host genes are involved in the regulation of several biological processes. For instance, *CDR1as, circSMARCA5* and* circPPP1R12A* affect cancer progression through sponging miR-7, forming an R-loop with parental DNA, and producing peptides, respectively. **(D)** SnoRNAs** (***SNORA23* and* SNORA42*) are produced by splicing of the intronic region of the host genes, while sno-derived RNAs (sdRNAs) *sdRNA68* and *sdRNA104* are produced by FUS-mediated self-splicing of some snoRNAs. Both snoRNAs and sdRNAs are reported to alter the process of tumorigenesis in some cancers.

Similar to mRNAs, the majority of ncRNAs are produced by the splicing of primary transcripts (also known as host genes). In this section, we discuss the splicing mechanisms underlying the biogenesis of various ncRNAs, including lncRNAs, circRNA, snoRNAs and sno-derived RNAs (sdRNAs), and clarify their biological significance in tumorigenesis and progression (Figure 2, Table 1 and 2).

### AS of lncRNAs from pre-lncRNAs

LncRNAs are >200 nucleotide-long RNA transcripts that do not encode proteins. They are generated from the splicing of pre-lncRNAs (Figure 2A). AS of lncRNAs produces different isoforms of lncRNAs that might exert different functions in cancer 35-39. For example, a multi-exon lncRNA *PXN-AS1*, which is regulated by SFs MBML3 and DDX17, could be spliced into multiple isoforms in hepatocellular carcinoma (HCC) 35. MBNL3 promotes the inclusion of the exon 4 of *PXN-AS1* to produce *PXN-AS1-L*, which inhibits myeloid cell leukemia (Mcl)-mediated cell apoptosis in a *PXN*-dependent manner 35. Whereas DDX17 induces the retention of the intron 3 of* PXN-AS1* to produce a novel aberrant isoform, *PXN-AS1-IR3*, which promotes HCC metastasis by inducing *MYC* transcription activation 36.

Moreover, AS of lncRNA *PCAT19* also generates two isoforms, namely *PCAT19-short* and *PCAT19-long*, which exhibit reciprocal expression in pancreatic cancer (PCa) 37. The *PCAT19-long* isoform interacts with HNRNPAB to activate a subset of cell-cycle genes associated with PCa progression, such as CHEK1 and AURKB, while the *PCAT19-short* isoform possesses potential tumor suppressive function 37. In addition, *NEAT1*, a well-known oncogenic lncRNA, produces two isoforms,* NEAT1_1* and *NEAT1_2* 40, 41. Among these, *NEAT1_2* is significantly upregulated in papillary thyroid carcinoma (PTC) and non-small cell lung cancer (NSCLC) compared with that in noncancerous tissues 38, 39, 42. In PTC, *NEAT1_2* significantly promotes cell growth and metastasis by acting as a sponge of *miR-106b-5p* to derepress ATAD2 expression 39; while in NSCLC, RBM10 regulates AS of* NEAT1* to downregulate NEAT1_2 expression, ultimately affecting the invasion and metastasis of NSCLC by suppressing the activation of the PTEN/PI3K/AKT/mTOR signaling pathway 38.

### LncRNAs produced by AS of pre-mRNAs

Recent studies revealed that some pre-mRNAs are bifunctional and could serve as precursors of both mRNAs and lncRNAs 43, 44 (Figure 2B). For instance, *PNUTS* is a bifunctional pre-mRNA encoding both *PNUTS* mRNA and *lncRNA-PNUTS* 43. In breast cancer, the *PNUTS* mRNA switches to *lncRNA-PNUTS*, which serves as a competitive sponge for miR-205, thereby promoting tumor epithelial-mesenchymal transition (EMT) 43. In lung adenocarcinoma (LUAD), bifunctional *PD-L1* pre-mRNA produces *PD-L1-lnc,* a lncRNA isoform, in addition to *PD-L1* mRNA 44. *PD-L1-lnc* is induced by IFNγ and binds to MYC to enhance its transcriptional activity, consequently activating its downstream genes and promoting LUAD cell proliferation and invasion 44. Altogether, these findings uncovered the novel lncRNA-mediated functions of pre-mRNAs in cancer.

### CircRNAs produced by back-splicing

CircRNAs are closed-loop RNA molecules produced by back splicing of the parental genes, in which the downstream splice site is reversed and joined to the upstream splice site 10. Previously, circRNAs were regarded as accidental "splicing noise" or by-products with few biological functions. However, increasing evidence suggests that circRNAs exert vital roles, such as miRNAs sponging, transcription regulation, and peptides encoding 45-47 (Figure 2C). For instance, circRNA *CDR1as* sponges *miR-7* to enhance the stability of *miR-7* targets including *E2F3, CKAP4*, and *TGFBR2*, thus promoting tumor growth and progression 45, 48. Additionally, *CircSMARCA5*, derived from the back-splicing of exon 15 and exon 16 of* SMARCA5,* binds to the genomic location of *SMARCA5* to form an R-loop*,* which pauses transcription at exon 15 of *SMARCA5* and produces truncated nonfunctional proteins, thus increasing sensitivity to cisplatin chemotherapy of breast cancer 46. Moreover, *CircPPP1R12A* is generated by reverse splicing exon 24 and 25 of *PPP1R12A* pre-mRNA, and encodes a 73-amino acid peptide, called circPPP1R12A-73aa 47. CircPPP1R12A-73aa promotes proliferation and metastasis of colon cancer through activating the Hippo-YAP signaling pathway 47. Therefore, circRNAs produced by splicing exhibit significant effects associated with carcinogenesis.

### Intronic snoRNAs and sdRNAs produced by RNA splicing

The majority of snoRNAs are processed from the introns of snoRNA host genes (SNHGs) 49 (Figure 2D). For example, *SNORA23* is generated by the splicing of the intronic region of the* IPO-7* gene, and its elevated levels significantly promote cancer cell survival and invasion in pancreatic ductal adenocarcinoma 50. Furthermore, *SNORA42* is spliced from the intron 10 of the* KIAA0907* gene, and its elevated levels play oncogenic roles via driving the malignant phenotype in NSCLC cells 51. Therefore, snoRNAs spliced from SNHGs perform essential functions during tumor development.

A preprint by Plewka P et al. argued a novel molecular mechanism of snoRNA self-splicing (Figure 2D), in which snoRNAs* SNORD104* and *SNORD68* interacted with RNA-binding protein (RBP) FUS and were further spliced into smaller sdRNAs (*sdRNA104* and *sdRNA68*, respectively) 52. FUS-dependent *sdRNA68* and *sdRNA104* regulated the expression of two colorectal cancer (CRC)-promoting genes, including* KCNQ10T1-001* antisense transcript and *BRE* mRNA 52, 53. Therefore, sdRNAs, as spliced products of snoRNAs, play critical pathological functions during tumor proliferation and progression.

Collectively, a numerous of ncRNAs generated by RNA splicing serve as potential prognostic and therapeutic biomarkers, indicating potential candidate targets for tumor prevention and treatment.

## Regulation of alternative splicing by ncRNAs

**Figure 3:**
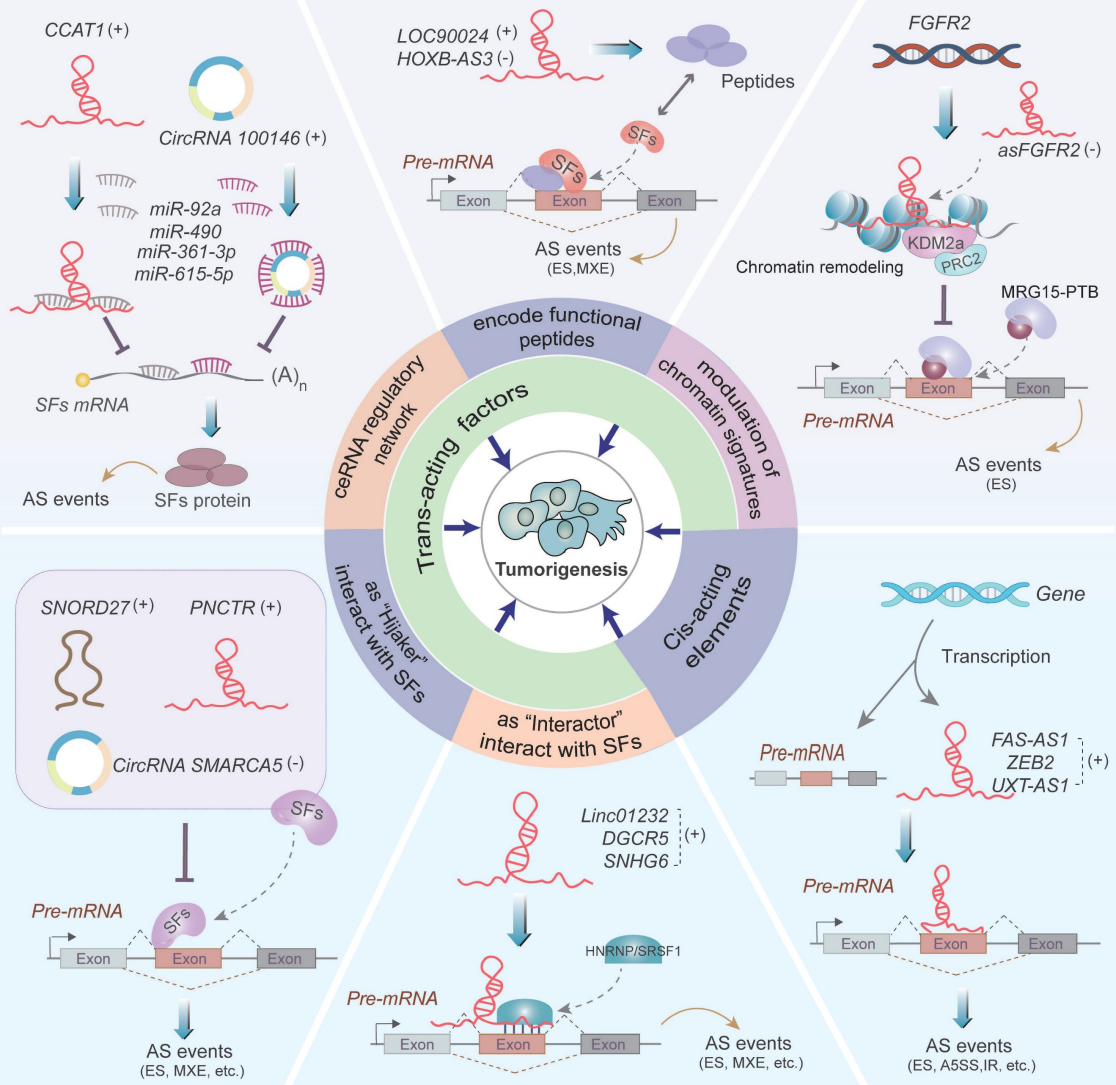
Regulation of alternative splicing by ncRNAs. Noncoding RNAs including lncRNAs, circRNAs, miRNAs, and snoRNAs regulate the occurrence of carcinogenesis-related AS events through two main splicing regulatory mechanisms. One way is to interact with the trans-acting factors, comprising acting as "Interactors" or "Hijackers" of SFs, participating in ceRNA regulatory network, encoding functional peptides and modulating chromatin signatures, all of which influence the expression and function of SFs. The second way is ncRNAs form dsRNA duplexes with the cis-acting elements, thus affecting tumorigenesis. The symbols (+) and (-) denote that ncRNAs act as oncogenes and tumor suppressor genes, respectively.

Recent studies have identified that ncRNAs exert their functions as regulators of AS events. As illustrated in Figure 3, ncRNAs regulate AS of pre-mRNAs by influencing the trans-acting factors or cis-acting elements, thus resulting in abnormal splicing of certain oncogenes or tumor suppressors (Table 1 and 2).

### NcRNAs regulate AS through influencing trans-acting factors

NcRNAs alter the expression and function of splicing factors (SFs) via interacting with SFs as “Interactors” or “Hijackers”, participating in competing endogenous RNA (ceRNA) regulatory network, encoding functional peptides and chromatin remodeling, thereby triggering tumor-associated alternative pre-mRNA splicing events.

#### NcRNAs acting as “Interactors” of SFs

NcRNAs recruit and stabilize SFs, and positively regulate the process of pre-mRNA splicing as “Interactors” of SFs, consequently influencing the pathogenesis process of tumorigenesis and progression 54-56 (Figure 3). Exemplified by linc01232, which significantly upregulates HNRNPA2B1 protein expression by suppressing its ubiquitin-mediated degradation in PCa cells 54. Subsequently, the stabilized HNRNPA2B1 participates in the AS of *A-Raf* pre-mRNA and facilitates the formation of full-length *A-Raf* (*A-Raf FL*) isoform, thus promoting PCa cells metastasis 54. Besides, lncRNA *DGCR5* indirectly regulates AS of *Mcl-1* pre-mRNA by interacting with serine- and arginine-rich splicing factor 1 (SRSF1) to increase its stability, and contributes to the generation of the full length of *Mcl-1* (*Mcl-1L*, anti-apoptotic isoform), thus facilitating the carcinogenesis of esophageal squamous cell carcinoma (ESCC) 55. Additionally, lncRNA *SNHG6* interacts with hnRNPA1, which triggers hnRNPA1-mediated splicing of *PKM* pre-mRNA and promotes the expression of PKM2 over PKM1, consequently enhancing aerobic glycolysis in CRC cells 56.

#### NcRNAs acting as “Hijackers” of SFs

Accumulating studies have supported that ncRNAs, including lncRNA, circRNAs and snoRNAs, function as "Hijackers", which competitively bind to SFs and thereby inhibit the interaction between SFs and target pre-mRNAs in the context of tumorigenesis and development 57-59 (Figure 3). For example, lncRNA *PNCTR* recruits many RBPs PTBP1 to the peri-nucleolar compartment and blocks its binding with *CHEK2* pre-mRNA 57. The interaction between PTBP1 and *CHEK2* pre-mRNA results in the upregulation of a CHEK2 isoform containing exon 8 and enhances the cell survival of cancer cells 57. Furthermore, it has been convincingly found that *circSMARCA5* hijacks SRSF1 and impedes the binding of SRSF1 protein to vascular endothelial growth factor A (*VEGFA*) pre-mRNA, thus reducing the ratio of pro-angiogenic (Iso8a) to anti-angiogenic (Iso8b) isoforms and inhibiting angiogenesis in glioblastoma multiforme (GBM) cells 58. In addition, *SNORD27* is a C/D box snoRNA that competitively binds to *U1* snRNAs and several SFs to form RNP complexes in HeLa cells 59. The RBPs facilitate the skipping of exon 12 in *E2F7* pre-mRNA and inhibit the inclusion of silent exon in *MAP4K3, ZBTB37, FER,* and* ABCA8* pre-mRNAs, thereby influencing E2F7-dependent cell cycle regulation 59.

#### NcRNAs alter SF expression by the ceRNA mechanism

The expression of SFs is regulated by various types of ncRNAs, which can functionally influence the outcomes of AS in cancer 60-62 (Figure 3). MiRNAs suppress SF expression by directly attaching to the 3'-untranslated region (3'-UTR) of the SF transcripts 60, 63. For instance, miR-92a reduces the expression of RNA-biding motif 4 (RBM4) by targeting the *RBM4* mRNA, which leads to elevated levels of exon 10-included nPTB transcript via an AS-coupled nonsense-mediated decay (NMD) mechanism 60. Subsequently, nPTB affects the splicing of *FGFR2* and *PKM2* and promotes the isoform *FGFR2* and *PKM2* of these two genes, respectively, thereby altering metabolic signature of CRC cells 60. More importantly, the ceRNA machinery can alter SF expression involving ncRNAs such as lncRNA, circRNAs, and pseudogenes, and these transcripts regulate each other through competing for shared miRNA regulators at the post-transcriptional level. Since microRNA response elements (MREs) exist on mRNAs, lncRNAs, circRNAs etc., and these RNAs can competitively sponge miRNAs by recognizing the same MREs 64-66. Thus, lncRNAs and circRNAs can functionally act as ceRNAs to sponge miRNAs and therefor modulate the expression of miRNA-targeted SF mRNAs 61, 62. Taking lncRNA *CCAT1* as an example, it sponges miR-490 and indirectly upregulates the expression of *hnRNPA1* and subsequently facilitates hnRNPA1-mediated AS events, leading to the migration and metastasis of gastric cancer 61. Similarly, *circRNA100146* directly sponges miR-361-3p and miR-615-5p and leads to promoting SF3B3 expression, consequently accelerating NSCLC cell proliferation and invasion through SF3B3-mediated AS regulation 62.

#### NcRNAs affect the function of SFs by encoding functional peptides

NcRNAs can encode "hidden" peptides that regulate RNA splicing 67, 68. As shown in Figure 3, *LOC90024* encodes a splicing regulatory small protein (SRSP) that enhances the binding of SRSF3 to the exon 3 of *Sp4* pre-mRNA, which induces the generation of long Sp4 isoform (encoding L-Sp4 protein), whereas suppresses short Sp4 isoform (encoding S-Sp4 peptide), ultimately promoting CRC tumorigenesis and progression 67. LncRNA *HOXB-AS3* encodes a conserved 53-aa peptide named HOXB-AS3, which competitively recognizes the arginine residues in the RGG motif of hnRNPA1 and antagonizes hnRNPA1-dependent *PKM* splicing, leading to the inhibition of PKM2 isoform and glucose metabolism in CRC cells 68. Taken together, these studies suggest that the ncRNAs-encoded functional peptides play important roles in AS regulation and may serve as novel targets for peptide-based anti-tumor drugs in the future.

#### NcRNAs alter the function of SFs by modulation of chromatin signatures

LncRNAs participate in the establishment of cell-specific splicing events by regulation of chromatin conformation signatures 69, 70. An antisense lncRNA, *asFGFR2,* recruits histone demethylase KDM2a and polycomb repressive complex 2 (PRC2) to the *FGFR2* locus creating an adverse chromatin environment that antagonizes the recruitment of splicing regulatory factor complex MRG15-PTB 69 (Figure 3). This regulatory mechanism eventually promotes the generation of exon IIIb-containing isoform *FGFR2 IIIb*, which inhibits tumorigenicity of HCC cells 69, 71. Besides, *ENST00000501665.2,* a splicing form of lncRNA* OIP5-AS1,* facilitates the interaction of chromatin-remodeling complexes SWI/SNF with the promoter of *OIP5* by directly binding to multiple nuclear RBPs including SMARCA4, a component of the SWI/SNF multi-subunit molecular complex, which leads to activated transcription and splicing of *OIP5* oncogene 70.

Altogether, these findings demonstrate that ncRNAs influence the expression and function of SFs to regulate multiple cancer-related AS events through different mechanisms, adding complexity into the ncRNAs-AS network.

### NcRNAs regulate AS via cis-acting elements

Cis-natural antisense transcripts (cis-NATs) are a new class of RNAs transcribed from the opposite strand of a coding gene and regulate gene expression by forming the double-stranded RNA (dsRNA) with the complementary region 72, 73. Cis-NATs can regulate AS of their antisense pre-mRNA, involved in diverse cellular functions during carcinogenesis 74-76 (Figure 3). Villamizar O et al. discovered that *FAS-AS1* (SAF), a cis-NAT transcribed from the antisense strand of* FAS*, interacts with *FAS* pre-mRNA to form RNA duplexes. These duplexes recruit SPF45 to mediate exon skipping of *FAS* and upregulate soluble Fas (sFas) protein to protect cells against FasL-induced apoptosis 74. Similarly, lncRNA *ZEB2 (Sip1)* interacts with the 5'-untranslated region (5'-UTR) of *ZEB2* to form a dsRNA 75. This interaction blocks the splicing of a large intron located in the 5'UTR of *ZEB2* which contains an internal ribosome entry site (IRES) critical for Zeb2 expression, thus promoting EMT of cancer cells through upregulating Zeb2 protein levels 75. Moreover, lncRNA *UXT-AS1,* transcribed from the antisense strand of *UXT*, binds to the cis-acting element within *UXT* pre-mRNA 76. The binding reduces the pro-apoptotic *UXT1* transcripts, meanwhile increases the pro-proliferative *UXT2* transcripts, thereby accelerating CRC progression 76. Altogether, these publications reveal a novel mechanism of ncRNAs-mediated regulation of AS in cancer cells by RNA duplex formation with the parental pre-mRNA.

## NcRNA-regulated AS events mediate drug resistance in oncology

**Figure 4:**
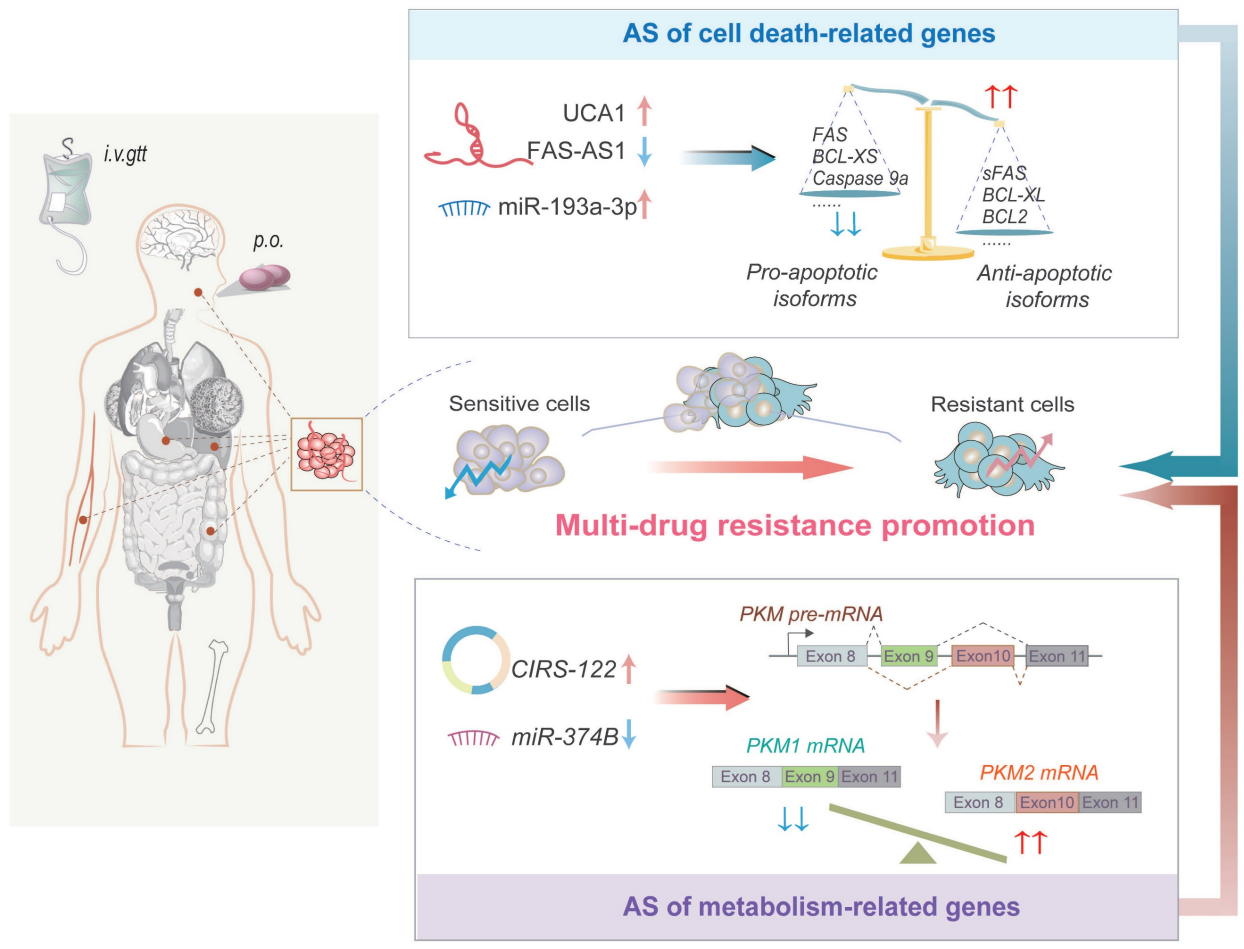
NcRNA-regulated AS events mediate drug resistance in oncology. Some lncRNAs, miRNAs and circRNAs impact drug resistance of cancer cells by triggering AS of cell death-related genes and metabolism-related genes. AS of cell death-related genes decreases the ratio of pro-/anti-apoptotic splicing isoforms to promote apoptotic tolerance in tumor cells, while AS of metabolism-related genes causes the upregulation of *PKM2* expression to promote glycolysis in cancer cells.

Chemotherapy and targeted therapy are frequently used in cancer treatment. However, multi-drug resistance (MDR) continues to hinder the clinical effects of chemotherapy. AS provides an opportunity for the pro-oncogenes to gain a new function that facilitates cancer cells evade from chemotherapy 77. For instance, Androgen receptor splice variant 7 (ARV7) is associated with abiraterone resistance in castration-resistant prostate cancer 78. Besides, Δ16HER2 splice variant is associated with lapatinib resistance in breast cancer 79. Therefore, owing to the important roles and promising clinical value of AS in drug resistance, as well as above mentioned extensive interplay between ncRNAs and AS, we summarized the interactive network of ncRNA-AS in drug resistance. This may provide new insights into understanding the MDR mechanism and identifying novel targets for preventing or reversing drug resistance in cancers (Figure 4 and Table 3).

### NcRNAs trigger AS of cell death-related genes to mediate chemoresistance

Apoptosis-evading cancer cells have a critical role in chemoresistance. NcRNAs regulate the AS events of apoptosis-related genes such as the *BCL-2* family, pro-apoptotic caspases, and *FAS*, thus altering the process of apoptosis and affecting the efficacy of chemotherapeutic drugs 80-82 (Figure 4). LncRNA *UCA1* is abnormally upregulated in cisplatin-resistant oral squamous cell carcinoma (OSCC) cells and sponges miR-184 to enhance SF1 expression. This augments SF1-mediated splicing of *BCL-2* family genes containing *Mcl-1* and increases BCL2 protein expression, thereby preventing apoptosis 80, 83, 84. Furthermore, *miR-193a-3p* interacts with SRSF2 to increase the anti-apoptotic variant of *BCL-X* (*BCL-XL*) and decrease the pro-apoptotic variant of *caspase 9* (*caspase 9a*), leading to cisplatin resistance in CD44^+^ gastric cancer cells 81. Interestingly, EZH2, the catalytic subunit of the PRC2 involved in H3K27 methylation, hyper-methylates the lncRNA *FAS-AS1* promoter and represses the FAS-AS1 expression in chemo-resistant B-cell lymphoma 82. The reduced FAS-AS1 expression causes increasing of soluble Fas receptor (sFAS) in a RBM5-dependent manner, which further suppresses apoptosis and leads to acquirement of chemoresistance 82, 85.

Autophagy is another fundamental mechanism that affects the sensitivity of cancer cells to anticancer agents, inhibition of which can also trigger apoptosis in cancer cells 86. Increasing studies have indicated that the interplay of ncRNAs-AS mediates drug resistance in cells by regulating autophagy 87, 88. For example, lncRNA *CRNDE* interacts with SRSF6 protein and reduces its stability, thus reducing AS of* PICALM* and inhibiting S-to-L isoform switch 87. Finally, *CRNDE* suppresses 5-FU/oxaliplatin resistance via attenuating autophagy flux in gastric cancer cells 87. Another study suggested that estrogen inhibits lncRNA *EGOT* in a dose-dependent manner in breast cancer 88. Low expression of *EGOT* interferes with pre-ITPR1/EGOT dsRNA formation and hnRNPH1 recruitment, consequently reducing autophagosome accumulation by downregulating ITPR1 protein, and ultimately enhancing paclitaxel resistance 88.

### NcRNAs trigger AS of metabolism-related genes to facilitate chemo- and targeted resistance

Cancer cells depend on aerobic glycolysis for ATP production and promoting rapid growth, which are also critical for the development of chemo- and targeted resistance 89. *PKM* is a critical enzyme in glycolysis metabolism, its splicing is crucial for metabolic regulation 90. AS of *PKM* pre-mRNA generates two isoforms, *PKM1* and *PKM2*, containing exon 9 and exon 10, respectively. Interestingly, *PKM2* encodes a protein that can catalyze glycolysis, and is correlated with poor prognosis in cancer patients, whereas *PKM1* encodes a protein that can promote oxidative phosphorylation 91-93. Hence, aberrant AS of *PKM* pre-mRNA triggered by ncRNAs can alter the sensitivity of cancer cells to chemotherapeutic agents 94, 95 (Figure 4). A study found that hnRNPA1 can bind to the flanking sequence of exon 9, resulting in exon 10 inclusion and thus sustaining a high PKM2/PKM1 ratio 96. However, miR-374b represses hnRNPA1 expression through targeting its 3'UTR, subsequently inhibiting *PKM2* and glycolysis to re-sensitize sorafenib-resistant HCC cells 94. It seems beyond dispute that AS of *PKM* regulated by miR-374b overexpression plays a significant role in overcoming sorafenib resistance in HCC. Another study indicated that *CIRS122* (hsa_circ_0005963) produced by back splicing upregulates the expression of *PKM2* by sponging miR-122, which stimulates glycolysis and leads to oxaliplatin resistance in CRC cells 95. This intercellular signal delivery could thus be used as a potential strategy for treating oxaliplatin-resistant CRC 95. Overall, these findings support that ncRNAs promote drug resistance in tumor cells by enhancing *PKM2*-mediated glycolysis, highlighting their potential as targets to reverse drug resistance in cancer cells.

## Targeting AS of ncRNAs or AS-related ncRNAs for cancer therapy

**Figure 5:**
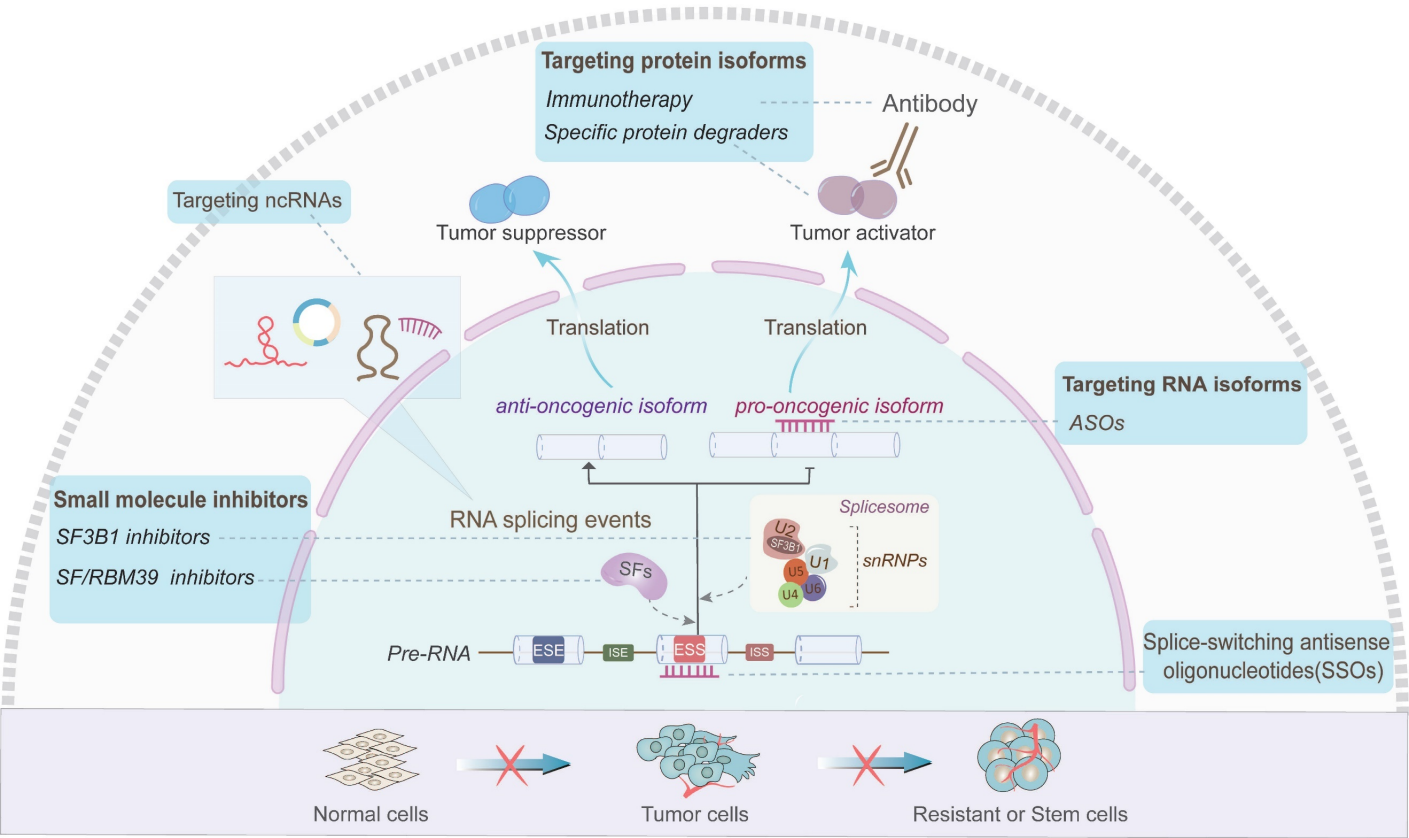
Strategies targeting AS of ncRNAs or AS-related ncRNAs for cancer therapy. Several antitumor strategies have been developed to alter AS events at distinct levels and consequently reverse the course of drug-resistant cells or carcinogenesis. These strategies include small molecule inhibitors of targeting SFs (such as RBM39) and splicesomal components (such as SF3B1), splice-switching antisense oligonucleotides (SSOs) targeting splicing "switch", antisense oligonucleotides (ASOs) targeting specific RNA isoforms, specific antibodies and protein degraders targeting oncogenic protein variants, and RNA therapies targeting AS-related ncRNAs.

Aberrant AS events are emerging as additional hallmarks of various cancers 33, 97-101. Currently, strategies targeting AS, such as small molecule targeting SFs or spliceosome, splice-switching antisense oligonucleotides (SSOs) and RNA or protein isoforms have exhibited potential values in clinical treatment (Figure 5). H3B-8800 is an orally available small molecule targeting SF3B1, which has entered phase I of clinical trials for the treatment of hematological malignancies 102, 103. Besides, SSOs are typically synthetic short-stranded RNAs that are designed to base-pair with cis-acting elements of target pre-mRNA, thus facilitating the conversion of splicing isoforms via blocking the binding of SFs to the pre-mRNA 104. Li et al. found that *BCL-X* SSO targeting the exon 2 of *BCL-X* pre-mRNA significantly elevates the *BCL-XS*/*BCL-XL* ratio and promotes glioma cell apoptosis 105. In addition, antisense oligonucleotides (ASOs) targeting RNA isoforms are also emerging as pharmacological agents 106. Li et al. identified an oncogenic lncRNA AC104041.1, and designed LNA-modified ASO targeting two splice variants of AC104041.1 which exhibited potent anti-tumor activity for head and neck squamous carcinoma (HNSCC) 107. Moreover, alternative tumor-specific antigens (TSAs) have recently been evaluated and considered to be bona fide targets of anti-cancer immunity 108, 109. Volpe G et al. reported that in Philadelphia chromosome-positive hematological malignancies, novel BCR-ABL transcripts are generated by AS, whose translational products contain C-terminal amino acid sequence derived from the out of reading frame (OOF) of the *ABL* gene 110. The presence of OOF-peptide can stimulate specific cytotoxic T lymphocyte reaction, suggesting that the BCR-ABL-OOF isoforms may be novel neoantigens for chronic myeloid leukemia therapy 110. Some recent studies showed that small molecule inhibitors can also be used as a potential source of tumor antigens and can be used in immunotherapy 111, 112. Elizabeth A et al. found that triple-negative breast cancer (TNBC) cells produce many intron-retained double-strand RNAs when treated with H3B-8800 111. These new antigens in turn activate the antiviral immune response and further induce exogenous apoptosis 111. Moreover, Lu X et al. revealed that sulfonamide derivative, Indisulam (E7070), degrades RBM39 in a dose-dependent manner and induces new antigens in cancer cells, thereby stimulating anti-tumor immune response and enhancing the efficacy of immune checkpoint inhibitors 112. Additionally, agents that mediate splicing isoform-specific degradation have also been developed 113. For instance, the AR-V7 degrades both AR-V7 isoform and full-length AR for prostate cancer, while DT2216 degrades the anti-apoptotic splicing isoform BCL-XL for liquid and solid cancers 114. Therefore, these small molecules have shown promising effects of reducing the tumor burden in a variety of cancers 114.

In addition to these AS-targeting strategies, ncRNAs as novel splicing regulators also hold new promise in cancer therapeutic (Figure 5). In B-cell lymphoma, 3-Deazaneplanocin A (DZNep) and ibrutinib could inhibit EZH-mediated methylation of lncRNA* FAS-AS1* promoter and upregulate *FAS-AS1*, thus enhancing FAS-mediated apoptosis of cancer cells 82, 115, 116. These findings indicate that *FAS-AS1* might be a promising target for lymphoma treatment and provide a rationale for the synergistic combination of EZH inhibitors and chemotherapy for lymphoma treatment.

## Conclusions and perspectives

Previously studies focused on AS related-coding genes, yet ncRNA-associated AS events have gained increasing interest, especially in the field of tumor epigenetics. This review highlights the interaction between ncRNAs and AS in cancer and summarizes distinct types of ncRNAs-mediated mechanisms involved in the aberrant regulation of AS events in various cancers. Owing to the extensive interactions between ncRNAs and AS and their mutual influence on cancer progression, AS-related ncRNAs have emerged as predictive biomarkers of chemotherapy and as potential targets for combination therapy. Hence, we also discussed the interplay of ncRNAs and AS in drug-resistant cells and the recent developments in cancer therapies targeting AS or AS-related ncRNAs.

Currently, researches on ncRNAs-AS network are conducted at a single gene level, since original papers usually performed rescue experiments to prove the concept. However, this simple model of single gene cannot fully reflect the complexity of the ncRNAs-AS interactive network or explain their important roles in cancers. Therefore, the mechanisms and functions of ncRNAs-AS network need to be further investigated, especially using bioinformatics to identify AS isoforms or AS-related ncRNAs that serve as prognostic biomarkers or therapeutic targets 117, 118. Recently, Deng et al. developed a comprehensive database LncAS2Cancer, which provides information regarding AS of lncRNAs across human cancers, as well as predicts the potential interaction between lncRNA and AS in cancers 118. However, to systematically explore the interplay between ncRNAs and AS, the development of high through-put sequencing methods detecting ncRNA-pre-mRNA or ncRNA-SF interaction is demanded. Nevertheless, the evidence to date is sufficient to demonstrate the importance of ncRNA-AS interplay in cancer. The development of effective drugs or strategies to target AS events of ncRNAs or AS-related ncRNAs, and their combination with current chemo-, targeted-, or immuno- therapies hold the promise for combating cancer in the future.

## Figures and Tables

**Table 1:** LncRNAs involved in the dysregulated alternative splicing events for carcinogenesis and progression

LncRNA	Mechanism	Target genes	Patterns	Biology function	Related diseases or processes	Refs
PXN-AS1	interacts with SF MBNL3	pre-PXN-AS1 → PXN-AS1-L	ES: exon 4 (-)	promotes proliferation and tumorigenesis	Hepatocellular carcinoma	35
	interacts with DDX17	pre-PXN-AS1 → PXN-AS1-IR3	IR: intron 3	activates MYC pathway	Hepatocellular carcinoma	36
PCAT19	produces long isoform that interacts with HNRNPAB	pre-PCAT19 → PCAT19-Long	/	activate a subset of cell-cycle genes	Prostate cancer	37
NEAT1	produces two isoforms and the long has tumor-promoting effect	pre-NEAT1 → NEAT1_2	/	promotes cell growth and metastasis	Non-small cell lung cancer, Papillary thyroid cancer	38, 39
lncRNA-PNUTS	produced by hnRNPE1-mediated AS of PUNTS	PNUTS pre-mRNA → lncRNA-PNUTS	A3SS (exon 12)	promotes tumor progression	Breast cancer, Epithelial-Mesenchymal Transition	43
PD-L1-lnc	produced by AS of PD-L1	PD-L1 pre-mRNA → PD-L1-lnc	/	enhances c-Myc transcriptional activity	Lung adenocarcinoma	44
Linc01232	suppresses the degradation of HNRNPA2B1	A-Raf pre-mRNA → A-Raf FL	ES (-)	activates MAPK/ERK signaling pathway	Pancreatic cancer	54
DGCR5	binds with SRSF1 to increase its stability	Mcl pre-mRNA → Mcl-1L	ES: exon 2 (-)	inhibits cell apoptosis	Esophageal squamous cell carcinoma	55
SNHG6	recruits and binds to hnRNPA1	PKM pre-mRNA → PKM2	MXE (exon 9,10)	enhances aerobic glycolysis	Colorectal cancer	56
PLANE	recruits and binds to hnRNPM	NCOR2 pre-mRNA → NCOR2-202 (-)	A5SS (intron 45)	promotes proliferation and tumorigenicity	Pan-cancer	119
BC200	recruits and binds to hnRNPA2B1	BCL-X pre-mRNA → BCL-XL	A5SS (exon 2) (-)	promotes cell proliferation	Breast cancer, Apoptosis	120
LincRNA-uc002yug.2	binds to SRSF1 and MBNL	RUNX1 pre-mRNA → RUNX1a	/	promotes cell proliferation and tumor growth	Esophageal cancer	121
PNCTR	recruits RBPs PTBP1and down-regulates them	CHEK2 pre-mRNA → CHEK (-)	ES: exon 8 (-)	promotes cell survival	Pan-cancer	57
TPM1-AS	combines competitively with RBM4	TPM1 pre-mRNA → TPM1 V2 and V7 (-)	MXE (exons 2a,2b)	inhibits cancer cell migration and formation of filopodia	Esophageal cancer	122
KASRT	interacts with SRSF1and down-regulates it	KLF6 pre-mRNA → KLF6-SV1	IR (-)	modulates P21/CCND1 pathway	Osteosarcoma	123
LINC01133	combines competitively with SRSF6	/	/	inhibits epithelial-mesenchymal transition and metastasis	Colorectal cancer	124
lncRNA AB074169 (lncAB)	combines competitively with RBP KHSRP and decreases its expression	p21 pre-mRNA→ p21 (CDKN1a) (-)	/	inhibits cell proliferation and tumor growth	Papillary thyroid cancer	125
CCAT1	targets miR-490 and up-regulates hnRNPA1 expression	/	/	promotes cell migration	Gastric cancer	61
LOC90024	encodes SRSP which interacts with SRSF3	Sp4 pre-mRNA → L-Sp4	ES: exon 3 (-)	promotes cancer tumorigenesis and progression	Colorectal cancer	67
HOXB-AS3	encodes HOXB-AS3 peptide which competitively binds to hnRNPA1	PKM pre-mRNA → PKM2 (-)	MXE (exon 9,10)	regulates cancer metabolism reprogramming	Colorectal cancer	68
asFGFR2	creates a chromatin environment and inhibits SF MRG15-PTB binding	FGFR2 pre-mRNA → FGFR2-IIIb	ES: exon IIIb (-)	suppresses cell proliferation and migratory potential	Hepatocellular carcinoma	69, 71
ENST00000501665.2	binds to RBPs of SWI/SNF chromatin remodeling complex	OIP5 pre-mRNA →OIP5	/	enhances expression of the oncogene	HEK293 cell	70
Fas-AS1	forms RNA-RNA duplexes with Fas pre-mRNA and recruits SPF45	Fas pre-mRNA → sFas	ES: exon 6	inhibits cell apoptosis	Apoptosis	74
ZEB2	forms RNA-RNA duplexes with ZEB2 pre-mRNA	ZEB2 pre-mRNA → ZEB2	IR	downregulates E-cadherin expression	Epithelial-Mesenchymal Transition	75
UXT-AS1	forms RNA-RNA duplexes with UXT pre-mRNA	UXT pre-mRNA → UXT2	A5SS	promotes cell proliferation and inhibits cell apoptosis	Colorectal cancer	76

* (-) refers to down-regulated AS products of target genes or suppressed AS events.

**Table 2:** CircRNAs, miRNAs and snoRNAs involved in the dysregulated alternative splicing events in cancer

Type	NcRNA	Mechanism	Target genes	Patterns	Biology function	Related diseases or processes	Refs
CircRNA	CircSMARCA5	combines competitively with SRSF1	VEGFA pre-mRNA → Iso8a (-)	ES: exon 8	inhibits angiogenesis	Glioblastoma multiforme	58
	CircURI1	combines competitively with hnRNPM	VEGFA pre-mRNA → VEGFAe7IN (-)	ES: exon 7	suppresses cell migration	Gastric cancer	126
	CircMYH9	combines competitively with hnRNPA2B1	p53 pre-mRNA → p53 (-)	/	promotes tumor growth by modulating Serine and glycine metabolism and redox homeostasis	Colorectal cancer	127
	CIRC-UBR5	recruits splicing regulatory factor QKI, NOVA1 and U1 SnRNA	/	/	regulates tumor differentiation	Non-small cell lung cancer	128
	CircRNA100146	targets miR-361-3p, miR-615-5p and up-regulates SF3B3 expression	/	/	promotes cell proliferation and invasion	Non-small cell lung cancer	62
MiRNA	miR-92a	reduces RBM4 expression	PTB pre-mRNA → nPTB	ES: exon 10 (-)	increases the invasion, migration, and mitochondrial activity	Colorectal cancer	60
	miR-193a-3p	reduces SRSF2 expression	BCL-X, caspase 9 pre-mRNA → BCL-XL, caspase 9a	A5SS (exon 2); ES (-)	promotes cisplatin resistance	Gastric cancer	81
	miR-30a-5p, miR-181a-5p, miR-216b-5p	decreases SRSF7 expression	SPP1 pre-mRNA → SPP1-c	MXE (exon 3,5)	decreases cell proliferation rate	Renal cancer	129
	miR-30c	decreases SRSF1 expression	/	/	suppresses cell survival and proliferation	Prostate cancer	130
	miR-1296	reduces SFPQ expression	/	/	promotes cell proliferation, invasion, migration	Colorectal cancer	131
	miR-133b	reduces SF3B4 expression	/	/	promotes cell proliferation and metastasis	Hepatocellular carcinoma	132
SnoRNA	SNORD104, SNORD68	interacts with RBP FUS	pre-snoRNAs → sdRNAs (sdRNA104, sdRNA68)	/	affects cell growth and proliferation	Colorectal cancer	52
	SNORD27	combines competitively with U1 snRNA and SFs	E2F7 pre-mRNA → E2F7	ES (exon 12)	regulates E2F7-dependent cell cycle regulation	Cell cycle	59
	SNORA70E	/	PARPBP-88 pre-mRNA → PARPBP-15	ES (exon 4)	promotes tumorigenesis and progression	Ovarian cancer	133

* (-) refers to down-regulated AS products of target genes or suppressed AS events.

**Table 3:** NcRNA-regulated AS events involved in drug resistance in cancer

NcRNA	Mechanism	Target genes	Biology function	Drugs	Related diseases	Refs
UCA1	targets miR-184 and up-regulates SF1 expression	Mcl pre-mRNA → Mcl-1L	increases BCL2 protein expression	Cisplatin	Oral squamous cell carcinoma	80, 83
miR-193a-3p	reduces SRSF2 expression	BCL-X, caspase 9 pre-mRNA → BCL-XL, caspase 9a	suppresses cell apoptosis	Cisplatin	Gastric cancer	81
Fas-AS1	low levels of it promote RBM5-mediated AS	Fas pre-mRNA → sFas	causes impaired Fas signaling in chemoresistance	Cytotoxic drugs	B-cell lymphoma	82, 85
CRNDE	interacts with SRSF6 and reduces its stability	PICALM pre-mRNA → PICALML (-)	reduces autophagy flux	5-FU/oxaliplatin	Gastric cancer	87
EGOT	forms pre-ITPR1/EGOT dsRNA and recruits hnRNPH1	ITPR1 pre-mRNA → ITPR1	promotes autophagy to increase drug sensitivity	Paclitaxel	Breast cancer	88
miR-374B	decreases hnRNPA1 expression	PKM pre-mRNA → PKM2 (-)	antagonizes PKM2-mediated glycolysis pathway	Sorafenib	Hepatocellular carcinoma	94
CIRS-122	targets miR-12	PKM pre-mRNA → PKM2	promotes glycolysis	Oxaliplatin	Colorectal cancer	95

*(-) refers to down-regulated AS products of target genes.

## Abbreviations

AS: Alternative splicing
NcRNAs: Non-coding RNAs
SR: Serine/arginine-rich
pre-mRNA: precursor messenger RNA
RNP: Ribonucleoprotein
snRNP: Small nuclear ribonucleoprotein
SF: Splicing factor
hnRNP: Heterogeneous nuclear ribonucleoprotein
SRE: Splicing regulatory element
ss: splice site
BPS: Branch point site
PPT: Polypyrimidine tract
ISE: Intronic splicing enhancer
ISS: Intronic splicing silencer
ES: Exon skipping
ESE: Exonic splicing enhancer
ESS: Exonic splicing silencer
A5SS: Alternative 5' splice site
A3SS: Alternative 3' splice site
IR: Intron retention
MXE: Mutually exclusive exon
LncRNA: Long non-coding RNA
miRNA: MicroRNA
circRNA: Circular RNA
snRNA: Small nuclear RNA
snoRNA: Small nucleolar RNA
PTC: Papillary thyroid carcinoma
HCC: hepatocellular carcinoma
EMT: Epithelial-mesenchymal transition
LUAD: Lung adenocarcinoma
SdRNA: SnoRNA-derived RNA
SNHG: SnoRNA host gene
ceRNA: Competing endogenous RNA
SRSF1: Serine- and arginine-rich splicing factor 1
RBP: RNA-binding protein
3'-UTR: 3'-untranslated region
RBM4: RNA-binding motif 4
MREs: microRNA response elements
NSCLC: Non-small cell lung cancer
CRC: Colorectal cancer
Pancreatic cancer: PCa
ESCC: Esophageal squamous cell carcinoma
GBM: Glioblastoma multiforme
NMD: Nonsense- mediated decay
SRSP: Splicing regulatory small protein
PRC2: Polycomb repressive complex 2
dsRNA: Double-stranded RNA
cis-NAT: cis-natural antisense transcript
MDR: Multi-drug resistance
ARV7: Androgen receptor splice variant 7
OSCC: Oral squamous cell carcinoma
HNSC: Head and neck squamous carcinoma
TNBC: Triple-negative breast cancer
OOF: Out of reading frame
FasL: Fas ligand
DZNep: 3-Deazaneplanocin A
SSO: Splice-switching antisense oligonucleotide
ASO: Antisense oligonucleotides

## References

[1] Lee TI, Young RA (2013). Transcriptional regulation and its misregulation in disease. Cell.

[2] Lee Y, Rio DC (2015). Mechanisms and Regulation of Alternative Pre-mRNA Splicing. Annu Rev Biochem.

[3] Wang E, Aifantis I (2020). RNA Splicing and Cancer. Trends Cancer.

[4] Baralle FE, Giudice J (2017). Alternative splicing as a regulator of development and tissue identity. Nat Rev Mol Cell Biol.

[5] Keren H, Lev-Maor G, Ast G (2010). Alternative splicing and evolution: diversification, exon definition and function. Nat Rev Genet.

[6] Blencowe BJ (2017). The Relationship between Alternative Splicing and Proteomic Complexity. Trends Biochem Sci.

[7] Kapranov P, Cheng J, Dike S, Nix DA, Duttagupta R, Willingham AT (2007). RNA maps reveal new RNA classes and a possible function for pervasive transcription. Science.

[8] Boon RA, Jaé N, Holdt L, Dimmeler S (2016). Long Noncoding RNAs: From Clinical Genetics to Therapeutic Targets?. J Am Coll Cardiol.

[9] Zlotorynski E (2019). Insights into the kinetics of microRNA biogenesis and turnover. Nat Rev Mol Cell Biol.

[10] Zhang XO, Dong R, Zhang Y, Zhang JL, Luo Z, Zhang J (2016). Diverse alternative back-splicing and alternative splicing landscape of circular RNAs. Genome Res.

[11] Kiss T (2002). Small nucleolar RNAs: an abundant group of noncoding RNAs with diverse cellular functions. Cell.

[12] Saw PE, Xu X, Chen J, Song EW (2021). Non-coding RNAs: the new central dogma of cancer biology. Sci China Life Sci.

[13] Romero-Barrios N, Legascue MF, Benhamed M, Ariel F, Crespi M (2018). Splicing regulation by long noncoding RNAs. Nucleic Acids Res.

[14] Liu Y, Liu X, Lin C, Jia X, Zhu H, Song J (2021). Noncoding RNAs regulate alternative splicing in Cancer. J Exp Clin Cancer Res.

[15] Zhang Y, Qian J, Gu C, Yang Y (2021). Alternative splicing and cancer: a systematic review. Signal Transduct Target Ther.

[16] Khan MR, Wellinger RJ, Laurent B (2021). Exploring the Alternative Splicing of Long Noncoding RNAs. Trends Genet.

[17] Marima R, Francies FZ, Hull R, Molefi T, Oyomno M, Khanyile R (2021). MicroRNA and Alternative mRNA Splicing Events in Cancer Drug Response/Resistance: Potent Therapeutic Targets. Biomedicines.

[18] Berget SM, Moore C, Sharp PA (1977). Spliced segments at the 5' terminus of adenovirus 2 late mRNA. Proc Natl Acad Sci U S A.

[19] Chow LT, Gelinas RE, Broker TR, Roberts RJ (1977). An amazing sequence arrangement at the 5' ends of adenovirus 2 messenger RNA. Cell.

[20] Suran M (2020). Finding the tail end: The discovery of RNA splicing. Proc Natl Acad Sci U S A.

[21] Shenasa H, Hertel KJ (2019). Combinatorial regulation of alternative splicing. Biochim Biophys Acta Gene Regul Mech.

[22] Padgett RA, Konarska MM, Grabowski PJ, Hardy SF, Sharp PA (1984). Lariat RNA's as intermediates and products in the splicing of messenger RNA precursors. Science.

[23] Ruskin B, Krainer AR, Maniatis T, Green MR (1984). Excision of an intact intron as a novel lariat structure during pre-mRNA splicing *in vitro*. Cell.

[24] Wahl MC, Will CL, Lührmann R (2009). The spliceosome: design principles of a dynamic RNP machine. Cell.

[25] Wilkinson ME, Charenton C, Nagai K (2020). RNA Splicing by the Spliceosome. Annu Rev Biochem.

[26] Wan R, Bai R, Zhan X, Shi Y (2020). How Is Precursor Messenger RNA Spliced by the Spliceosome?. Annu Rev Biochem.

[27] Hoskins AA, Friedman LJ, Gallagher SS, Crawford DJ, Anderson EG, Wombacher R (2011). Ordered and dynamic assembly of single spliceosomes. Science.

[28] Fu XD (2004). Towards a splicing code. Cell.

[29] Busch A, Hertel KJ (2012). Evolution of SR protein and hnRNP splicing regulatory factors. Wiley Interdiscip Rev RNA.

[30] Fu XD, Ares M Jr (2014). Context-dependent control of alternative splicing by RNA-binding proteins. Nat Rev Genet.

[31] Paronetto MP, Passacantilli I, Sette C (2016). Alternative splicing and cell survival: from tissue homeostasis to disease. Cell Death Differ.

[32] Gallego-Paez LM, Bordone MC, Leote AC, Saraiva-Agostinho N, Ascensão-Ferreira M, Barbosa-Morais NL (2017). Alternative splicing: the pledge, the turn, and the prestige: The key role of alternative splicing in human biological systems. Hum Genet.

[33] Zhang J, Manley JL (2013). Misregulation of pre-mRNA alternative splicing in cancer. Cancer Discov.

[34] Black DL (2003). Mechanisms of alternative pre-messenger RNA splicing. Annu Rev Biochem.

[35] Yuan JH, Liu XN, Wang TT, Pan W, Tao QF, Zhou WP (2017). The MBNL3 splicing factor promotes hepatocellular carcinoma by increasing PXN expression through the alternative splicing of lncRNA-PXN-AS1. Nat Cell Biol.

[36] Zhou HZ, Li F, Cheng ST, Xu Y, Deng HJ, Gu DY (2022). DDX17-regulated alternative splicing that produced an oncogenic isoform of PXN-AS1 to promote HCC metastasis. Hepatology.

[37] Hua JT, Ahmed M, Guo H, Zhang Y, Chen S, Soares F (2018). Risk SNP-Mediated Promoter-Enhancer Switching Drives Prostate Cancer through lncRNA PCAT19. Cell.

[38] Cong S, Di X, Li R, Cao Y, Jin X, Tian C (2022). RBM10 regulates alternative splicing of lncRNA Neat1 to inhibit the invasion and metastasis of NSCLC. Cancer Cell Int.

[39] Sun W, Lan X, Zhang H, Wang Z, Dong W, He L (2018). NEAT1_2 functions as a competing endogenous RNA to regulate ATAD2 expression by sponging microRNA-106b-5p in papillary thyroid cancer. Cell Death Dis.

[40] Clemson CM, Hutchinson JN, Sara SA, Ensminger AW, Fox AH, Chess A (2009). An architectural role for a nuclear noncoding RNA: NEAT1 RNA is essential for the structure of paraspeckles. Mol Cell.

[41] Sasaki YT, Ideue T, Sano M, Mituyama T, Hirose T (2009). MENepsilon/beta noncoding RNAs are essential for structural integrity of nuclear paraspeckles. Proc Natl Acad Sci U S A.

[42] Lan X, Zhang H, Wang Z, Dong W, Sun W, Shao L (2015). Genome-wide analysis of long noncoding RNA expression profile in papillary thyroid carcinoma. Gene.

[43] Grelet S, Link LA, Howley B, Obellianne C, Palanisamy V, Gangaraju VK (2017). A regulated PNUTS mRNA to lncRNA splice switch mediates EMT and tumour progression. Nat Cell Biol.

[44] Qu S, Jiao Z, Lu G, Yao B, Wang T, Rong W (2021). PD-L1 lncRNA splice isoform promotes lung adenocarcinoma progression via enhancing c-Myc activity. Genome Biol.

[45] Zhong Q, Huang J, Wei J, Wu R (2019). Circular RNA CDR1as sponges miR-7-5p to enhance E2F3 stability and promote the growth of nasopharyngeal carcinoma. Cancer Cell Int.

[46] Xu X, Zhang J, Tian Y, Gao Y, Dong X, Chen W (2020). CircRNA inhibits DNA damage repair by interacting with host gene. Mol Cancer.

[47] Zheng X, Chen L, Zhou Y, Wang Q, Zheng Z, Xu B (2019). A novel protein encoded by a circular RNA circPPP1R12A promotes tumor pathogenesis and metastasis of colon cancer via Hippo-YAP signaling. Mol Cancer.

[48] Guo Z, Cao Q, Zhao Z, Song C (2020). Biogenesis, Features, Functions, and Disease Relationships of a Specific Circular RNA: CDR1as. Aging Dis.

[49] Filipowicz W, Pogacić V (2002). Biogenesis of small nucleolar ribonucleoproteins. Curr Opin Cell Biol.

[50] Cui L, Nakano K, Obchoei S, Setoguchi K, Matsumoto M, Yamamoto T (2017). Small Nucleolar Noncoding RNA SNORA23, Up-Regulated in Human Pancreatic Ductal Adenocarcinoma, Regulates Expression of Spectrin Repeat-Containing Nuclear Envelope 2 to Promote Growth and Metastasis of Xenograft Tumors in Mice. Gastroenterology.

[51] Mei YP, Liao JP, Shen J, Yu L, Liu BL, Liu L (2012). Small nucleolar RNA 42 acts as an oncogene in lung tumorigenesis. Oncogene.

[52] Plewka P, Szcześniak M, Stepien A, Żywicki M, Pacak A, Colombo M (2018). FUS controls the processing of snoRNAs into smaller RNA fragments that can regulate gene expression. bioRxiv.

[53] Nakano S, Murakami K, Meguro M, Soejima H, Higashimoto K, Urano T (2006). Expression profile of LIT1/KCNQ1OT1 and epigenetic status at the KvDMR1 in colorectal cancers. Cancer Sci.

[54] Meng LD, Shi GD, Ge WL, Huang XM, Chen Q, Yuan H (2020). Linc01232 promotes the metastasis of pancreatic cancer by suppressing the ubiquitin-mediated degradation of HNRNPA2B1 and activating the A-Raf-induced MAPK/ERK signaling pathway. Cancer Lett.

[55] Duan Y, Jia Y, Wang J, Liu T, Cheng Z, Sang M (2021). Long noncoding RNA DGCR5 involves in tumorigenesis of esophageal squamous cell carcinoma via SRSF1-mediated alternative splicing of Mcl-1. Cell Death Dis.

[56] Lan Z, Yao X, Sun K, Li A, Liu S, Wang X (2020). The Interaction Between lncRNA SNHG6 and hnRNPA1 Contributes to the Growth of Colorectal Cancer by Enhancing Aerobic Glycolysis Through the Regulation of Alternative Splicing of PKM. Front Oncol.

[57] Yap K, Mukhina S, Zhang G, Tan JSC, Ong HS, Makeyev EV (2018). A Short Tandem Repeat-Enriched RNA Assembles a Nuclear Compartment to Control Alternative Splicing and Promote Cell Survival. Mol Cell.

[58] Barbagallo D, Caponnetto A, Brex D, Mirabella F, Barbagallo C, Lauretta G (2019). CircSMARCA5 Regulates VEGFA mRNA Splicing and Angiogenesis in Glioblastoma Multiforme Through the Binding of SRSF1. Cancers (Basel).

[59] Falaleeva M, Pages A, Matuszek Z, Hidmi S, Agranat-Tamir L, Korotkov K (2016). Dual function of C/D box small nucleolar RNAs in rRNA modification and alternative pre-mRNA splicing. Proc Natl Acad Sci U S A.

[60] Liang YC, Lin WC, Lin YJ, Lin JC (2015). The impact of RNA binding motif protein 4-regulated splicing cascade on the progression and metabolism of colorectal cancer cells. Oncotarget.

[61] Zhou B, Wang Y, Jiang J, Jiang H, Song J, Han T (2016). The long noncoding RNA colon cancer-associated transcript-1/miR-490 axis regulates gastric cancer cell migration by targeting hnRNPA1. IUBMB life.

[62] Chen L, Nan A, Zhang N, Jia Y, Li X, Ling Y (2019). Circular RNA 100146 functions as an oncogene through direct binding to miR-361-3p and miR-615-5p in non-small cell lung cancer. Mol Cancer.

[63] Bartel DP (2004). MicroRNAs: genomics, biogenesis, mechanism, and function. Cell.

[64] Thomson DW, Dinger ME (2016). Endogenous microRNA sponges: evidence and controversy. Nat Rev Genet.

[65] Sen R, Ghosal S, Das S, Balti S, Chakrabarti J (2014). Competing endogenous RNA: the key to posttranscriptional regulation. ScientificWorldJournal.

[66] Alkan AH, Akgül B (2022). Endogenous miRNA Sponges. Methods Mol Biol.

[67] Meng N, Chen M, Chen D, Chen XH, Wang JZ, Zhu S (2020). Small Protein Hidden in lncRNA LOC90024 Promotes "Cancerous" RNA Splicing and Tumorigenesis. Adv Sci (Weinh).

[68] Huang JZ, Chen M, Chen D, Gao XC, Zhu S, Huang H (2017). A Peptide Encoded by a Putative lncRNA HOXB-AS3 Suppresses Colon Cancer Growth. Mol Cell.

[69] Gonzalez I, Munita R, Agirre E, Dittmer TA, Gysling K, Misteli T (2015). A lncRNA regulates alternative splicing via establishment of a splicing-specific chromatin signature. Nat Struct Mol Biol.

[70] Wanowska E, Kubiak M, Makałowska I, Szcześniak MW (2021). A chromatin-associated splicing isoform of OIP5-AS1 acts in cis to regulate the OIP5 oncogene. RNA Biol.

[71] Amann T, Bataille F, Spruss T, Dettmer K, Wild P, Liedtke C (2010). Reduced expression of fibroblast growth factor receptor 2IIIb in hepatocellular carcinoma induces a more aggressive growth. The American journal of pathology.

[72] Li K, Ramchandran R (2010). Natural antisense transcript: a concomitant engagement with protein-coding transcript. Oncotarget.

[73] Conley AB, Jordan IK (2012). Epigenetic regulation of human cis-natural antisense transcripts. Nucleic Acids Res.

[74] Villamizar O, Chambers CB, Riberdy JM, Persons DA, Wilber A (2016). Long noncoding RNA Saf and splicing factor 45 increase soluble Fas and resistance to apoptosis. Oncotarget.

[75] Beltran M, Puig I, Peňa C, García JM, Alvarez AB, Pena R (2008). A natural antisense transcript regulates Zeb2/Sip1 gene expression during Snail1-induced epithelial-mesenchymal transition. Genes Dev.

[76] Yin J, Luo W, Zeng X, Zeng L, Li Z, Deng X (2017). UXT-AS1-induced alternative splicing of UXT is associated with tumor progression in colorectal cancer. Am J Cancer Res.

[77] Sciarrillo R, Wojtuszkiewicz A, Assaraf YG, Jansen G, Kaspers GJL, Giovannetti E (2020). The role of alternative splicing in cancer: From oncogenesis to drug resistance. Drug Resist Updat.

[78] He Y, Lu J, Ye Z, Hao S, Wang L, Kohli M (2018). Androgen receptor splice variants bind to constitutively open chromatin and promote abiraterone-resistant growth of prostate cancer. Nucleic Acids Res.

[79] Tilio M, Gambini V, Wang J, Garulli C, Kalogris C, Andreani C (2016). Irreversible inhibition of Δ16HER2 is necessary to suppress Δ16HER2-positive breast carcinomas resistant to Lapatinib. Cancer Lett.

[80] Fang Z, Zhao J, Xie W, Sun Q, Wang H, Qiao B (2017). LncRNA UCA1 promotes proliferation and cisplatin resistance of oral squamous cell carcinoma by sunppressing miR-184 expression. Cancer Med.

[81] Lee SD, Yu D, Lee DY, Shin HS, Jo JH, Lee YC (2019). Upregulated microRNA-193a-3p is responsible for cisplatin resistance in CD44(+) gastric cancer cells. Cancer Sci.

[82] Sehgal L, Mathur R, Braun FK, Wise JF, Berkova Z, Neelapu S (2014). FAS-antisense 1 lncRNA and production of soluble versus membrane Fas in B-cell lymphoma. Leukemia.

[83] Palve V, Mallick S, Ghaisas G, Kannan S, Teni T (2014). Overexpression of Mcl-1L splice variant is associated with poor prognosis and chemoresistance in oral cancers. PLoS One.

[84] Ismail NZ, Md Saad S, Adebayo IA, Md Toha Z, Abas R, Mohamad Zain NN (2022). The antiproliferative and apoptotic potential of Clinacanthus nutans against human breast cancer cells through targeted apoptosis pathway. Environ Sci Pollut Res Int.

[85] Friesen C, Fulda S, Debatin KM (1999). Cytotoxic drugs and the CD95 pathway. Leukemia.

[86] Ho CJ, Gorski SM (2019). Molecular Mechanisms Underlying Autophagy-Mediated Treatment Resistance in Cancer. Cancers (Basel).

[87] Zhang F, Wang H, Yu J, Yao X, Yang S, Li W (2021). LncRNA CRNDE attenuates chemoresistance in gastric cancer via SRSF6-regulated alternative splicing of PICALM. Mol Cancer.

[88] Xu S, Wang P, Zhang J, Wu H, Sui S, Zhang J (2019). Ai-lncRNA EGOT enhancing autophagy sensitizes paclitaxel cytotoxicity via upregulation of ITPR1 expression by RNA-RNA and RNA-protein interactions in human cancer. Mol Cancer.

[89] Liberti MV, Locasale JW (2016). The Warburg Effect: How Does it Benefit Cancer Cells?. Trends Biochem Sci.

[90] Dayton TL, Jacks T, Vander Heiden MG (2016). PKM2, cancer metabolism, and the road ahead. EMBO Rep.

[91] Wang W, He Q, Sun J, Liu Z, Zhao L, Lu Z (2017). Pyruvate kinase M2 deregulation enhances the metastatic potential of tongue squamous cell carcinoma. Oncotarget.

[92] Mohammad GH, Olde Damink SW, Malago M, Dhar DK, Pereira SP (2016). Pyruvate Kinase M2 and Lactate Dehydrogenase A Are Overexpressed in Pancreatic Cancer and Correlate with Poor Outcome. PloS One.

[93] Chaneton B, Gottlieb E (2012). Rocking cell metabolism: revised functions of the key glycolytic regulator PKM2 in cancer. Trends Biochem Sci.

[94] Zhang M, Zhang H, Hong H, Zhang Z (2019). MiR-374b re-sensitizes hepatocellular carcinoma cells to sorafenib therapy by antagonizing PKM2-mediated glycolysis pathway. Am J Cancer Res.

[95] Wang X, Zhang H, Yang H, Bai M, Ning T, Deng T (2020). Exosome-delivered circRNA promotes glycolysis to induce chemoresistance through the miR-122-PKM2 axis in colorectal cancer. Mol Oncol.

[96] Chen M, David CJ, Manley JL (2012). Concentration-dependent control of pyruvate kinase M mutually exclusive splicing by hnRNP proteins. Nat Struct Mol Biol.

[97] Ladomery M (2013). Aberrant alternative splicing is another hallmark of cancer. Int J Cell Biol.

[98] Farina AR, Cappabianca L, Sebastiano M, Zelli V, Guadagni S, Mackay AR (2020). Hypoxia-induced alternative splicing: the 11th Hallmark of Cancer. J Exp Clin Cancer Res.

[99] Song X, Zeng Z, Wei H, Wang Z (2018). Alternative splicing in cancers: From aberrant regulation to new therapeutics. Semin Cell Dev Biol.

[100] Hanahan D, Weinberg RA (2011). Hallmarks of cancer: the next generation. Cell.

[101] Bonnal SC, López-Oreja I, Valcárcel J (2020). Roles and mechanisms of alternative splicing in cancer - implications for care. Nat Rev Clin Oncol.

[102] Seiler M, Yoshimi A, Darman R, Chan B, Keaney G, Thomas M (2018). H3B-8800, an orally available small-molecule splicing modulator, induces lethality in spliceosome-mutant cancers. Nat Med.

[103] Steensma DP, Wermke M, Klimek VM, Greenberg PL, Font P, Komrokji RS (2021). Phase I First-in-Human Dose Escalation Study of the oral SF3B1 modulator H3B-8800 in myeloid neoplasms. Leukemia.

[104] Havens MA, Hastings ML (2016). Splice-switching antisense oligonucleotides as therapeutic drugs. Nucleic Acids Res.

[105] Li Z, Li Q, Han L, Tian N, Liang Q, Li Y (2016). Pro-apoptotic effects of splice-switching oligonucleotides targeting Bcl-x pre-mRNA in human glioma cell lines. Oncol Rep.

[106] Rinaldi C, Wood MJA (2018). Antisense oligonucleotides: the next frontier for treatment of neurological disorders. Nat Rev Neurol.

[107] Li M, Ding X, Zhang Y, Li X, Zhou H, Yang L (2020). Antisense oligonucleotides targeting lncRNA AC104041.1 induces antitumor activity through Wnt2B/beta-catenin pathway in head and neck squamous cell carcinomas. Cell Death Dis.

[108] Smith CC, Selitsky SR, Chai S, Armistead PM, Vincent BG, Serody JS (2019). Alternative tumour-specific antigens. Nat Rev Cancer.

[109] Pan Y, Kadash-Edmondson KE, Wang R, Phillips J, Liu S, Ribas A (2021). RNA Dysregulation: An Expanding Source of Cancer Immunotherapy Targets. Trends Pharmacol Sci.

[110] Volpe G, Cignetti A, Panuzzo C, Kuka M, Vitaggio K, Brancaccio M (2007). Alternative BCR/ABL splice variants in Philadelphia chromosome-positive leukemias result in novel tumor-specific fusion proteins that may represent potential targets for immunotherapy approaches. Cancer Res.

[111] Bowling EA, Wang JH, Gong F, Wu W, Neill NJ, Kim IS (2021). Spliceosome-targeted therapies trigger an antiviral immune response in triple-negative breast cancer. Cell.

[112] Lu SX, De Neef E, Thomas JD, Sabio E, Rousseau B, Gigoux M (2021). Pharmacologic modulation of RNA splicing enhances anti-tumor immunity. Cell.

[113] Jan M, Sperling AS, Ebert BL (2021). Cancer therapies based on targeted protein degradation - lessons learned with lenalidomide. Nat Rev Clin Oncol.

[114] Mullard A (2021). Targeted protein degraders crowd into the clinic. Nat Rev Drug Discov.

[115] Burger JA, Chiorazzi N (2013). B cell receptor signaling in chronic lymphocytic leukemia. Trends Immunol.

[116] Cao Q, Yu J, Dhanasekaran SM, Kim JH, Mani RS, Tomlins SA (2008). Repression of E-cadherin by the polycomb group protein EZH2 in cancer. Oncogene.

[117] Kahles A, Lehmann KV, Toussaint NC, Huser M, Stark SG, Sachsenberg T (2018). Comprehensive Analysis of Alternative Splicing Across Tumors from 8,705 Patients. Cancer Cell.

[118] Deng Y, Luo H, Yang Z, Liu L (2021). LncAS2Cancer: a comprehensive database for alternative splicing of lncRNAs across human cancers. Brief Bioinform.

[119] Teng L, Feng YC, Guo ST, Wang PL, Qi TF, Yue YM (2021). The pan-cancer lncRNA PLANE regulates an alternative splicing program to promote cancer pathogenesis. Nat Commun.

[120] Singh R, Gupta SC, Peng WX, Zhou N, Pochampally R, Atfi A (2016). Regulation of alternative splicing of Bcl-x by BC200 contributes to breast cancer pathogenesis. Cell Death Dis.

[121] Wu H, Zheng J, Deng J, Zhang L, Li N, Li W (2015). LincRNA-uc002yug.2 involves in alternative splicing of RUNX1 and serves as a predictor for esophageal cancer and prognosis. Oncogene.

[122] Huang GW, Zhang YL, Liao LD, Li EM, Xu LY (2017). Natural antisense transcript TPM1-AS regulates the alternative splicing of tropomyosin I through an interaction with RNA-binding motif protein 4. Int J Biochem Cell Biol.

[123] Chen K, Li C, Huang S, Chen Y, Zhu X (2021). LncRNA KASRT Serves as a Potential Treatment Target by Regulating SRSF1-Related KLF6 Alternative Splicing and the P21/CCND1 Pathway in Osteosarcoma: An *In vitro* and *In vivo* Study. Front Oncol.

[124] Kong J, Sun W, Li C, Wan L, Wang S, Wu Y (2016). Long non-coding RNA LINC01133 inhibits epithelial-mesenchymal transition and metastasis in colorectal cancer by interacting with SRSF6. Cancer Lett.

[125] Gou Q, Gao L, Nie X, Pu W, Zhu J, Wang Y (2018). Long Noncoding RNA AB074169 Inhibits Cell Proliferation via Modulation of KHSRP-Mediated CDKN1a Expression in Papillary Thyroid Carcinoma. Cancer Res.

[126] Wang X, Li J, Bian X, Wu C, Hua J, Chang S (2021). CircURI1 interacts with hnRNPM to inhibit metastasis by modulating alternative splicing in gastric cancer. Proc Natl Acad Sci U S A.

[127] Liu X, Liu Y, Liu Z, Lin C, Meng F, Xu L (2021). CircMYH9 drives colorectal cancer growth by regulating serine metabolism and redox homeostasis in a p53-dependent manner. Mol Cancer.

[128] Qin M, Wei G, Sun X (2018). Circ-UBR5: An exonic circular RNA and novel small nuclear RNA involved in RNA splicing. Biochem Biophys Res Commun.

[129] Boguslawska J, Sokol E, Rybicka B, Czubaty A, Rodzik K, Piekielko-Witkowska A (2016). microRNAs target SRSF7 splicing factor to modulate the expression of osteopontin splice variants in renal cancer cells. Gene.

[130] Huang YQ, Ling XH, Yuan RQ, Chen ZY, Yang SB, Huang HX (2017). miR-30c suppresses prostate cancer survival by targeting the ASF/SF2 splicing factor oncoprotein. Mol Med Rep.

[131] Tao Y, Ma C, Fan Q, Wang Y, Han T, Sun C (2018). MicroRNA-1296 Facilitates Proliferation, Migration And Invasion Of Colorectal Cancer Cells By Targeting SFPQ. J Cancer.

[132] Liu Z, Li W, Pang Y, Zhou Z, Liu S, Cheng K (2018). SF3B4 is regulated by microRNA-133b and promotes cell proliferation and metastasis in hepatocellular carcinoma. EBioMedicine.

[133] Chen S, Li QH, Chen X, Bao HJ, Wu W, Shen F (2022). SNORA70E promotes the occurrence and development of ovarian cancer through pseudouridylation modification of RAP1B and alternative splicing of PARPBP. J Cell Mol Med.

